# Advances in Diagnostic Parameters and Surgical Techniques for Patellofemoral Instability and Extensor Mechanism Disorders: A Comprehensive Review

**DOI:** 10.7759/cureus.73254

**Published:** 2024-11-07

**Authors:** Alexandru-Mihai Papuc, Georgian L Iacobescu, Antonio-Daniel Corlatescu, Bogdan Serban, Razvan Spiridonica, Catalin Cirstoiu

**Affiliations:** 1 Orthopedics and Traumatology, University Emergency Hospital, Bucharest, ROU; 2 General Medicine, Carol Davila University of Medicine and Pharmacy, Bucharest, ROU; 3 Orthopedics and Trauma, Carol Davila University of Medicine and Pharmacy, Bucharest, ROU

**Keywords:** arthroscopic surgery, diagnostic imaging, mpfl reconstruction, patellar dislocation, patellofemoral instability

## Abstract

Extensor mechanism misalignment in the knee, particularly patellofemoral instability (PFI), requires detailed diagnostic and therapeutic approaches. This comprehensive review intends to present recent advancements in diagnostic imaging technologies as well as surgical approaches that have significantly advanced the management of this condition. An extensive literature review covered recent studies and clinical practices related to diagnosing and treating knee extensor mechanism misalignment, such as three-dimensional imaging technologies, arthroscopic surgical techniques, postoperative rehabilitation protocols, and others. Three-dimensional imaging technologies such as MRI and CT scans have revolutionized diagnosing and managing PFI by providing detailed assessments of anatomical anomalies, leading to personalized treatment plans tailored specifically for each individual's anatomy. Arthroscopic surgery, with techniques such as lateral release, medial patellofemoral ligament reconstruction, and tibial tubercle osteotomy, has become an efficient minimally invasive solution, with advancements like digitally calibrated measurement tools further improving surgical precision and outcomes. The combined use of advanced imaging technologies and surgical methods has greatly advanced the management of knee extensor mechanism misalignment. Personalized medicine approaches combined with minimally invasive surgical techniques have yielded promising recovery times and outcomes, and future directions include exploring biological therapies, improving rehabilitation protocols, and expanding diagnostic technologies to address misalignments more comprehensively. With modern diagnostic tools and innovative surgical techniques, more effective and personalized treatments for extensor mechanism misalignment in the knee have become possible. These advances could significantly enhance the quality of life for individuals suffering from PFI disorders; ongoing research collaboration should serve to optimize diagnosis, treatment, and prevention strategies further.

## Introduction and background

Medical professionals commonly see injuries to the extensor mechanism of the knee that are important for activities like walking and sports. These injuries can occur due to both traumatic and non-traumatic reasons. It is crucial to understand the anatomy of the knee extension, which includes the quadriceps muscles, the patellar tendon, and the patella itself. The quadriceps group, consisting of the rectus femoris, vastus lateralis, vastus intermedius, and vastus medialis, come together into the quadriceps tendon, which attaches to the upper part of the patella. The patella, located in the femoral trochlear groove, connects the quadriceps tendon to the patellar tendon. The patellar tendon runs from the lower part of the patella to the tibial tuberosity. This intricate structure is essential for effective knee extension, which is necessary for walking and various sports activities [1].

Medial and lateral retinacula, made up of quadriceps fibers reinforced by various ligaments, enhance the stability of the extensor mechanism. Fat pads and bursae within the knee also help reduce friction and facilitate movement. At the same time, blood supply comes from genicular arteries and innervation from tibial, femoral, obturator, and common peroneal nerves. Traumatic knee extensor mechanism injuries often occur when the quadriceps contract against a flexed knee during balance recovery from falls, missteps, jumping, or landing awkwardly on uneven terrain. They may also happen following minor falls due to preexisting health conditions. Symptoms include audible popping noises or tearing sensations, followed by pain in the knee joint area and an inability to bear weight. Quadriceps tears typically occur at the tendon insertion to the patella, leading to an inferiorly displaced patella (patella baja). Patellar tendon tears may involve avulsions from the lower pole of the patella, mid-tendon tears, or avulsions from tibial tubercle avulsions, causing a superiorly displaced patella (patella alta). Patellar fractures often result from direct trauma or forceful contraction and may be vertical, marginal, transverse, or comminuted in nature. Vertical fractures may not disrupt the extensor mechanism, while in marginal or transverse fractures, there is a possibility [2,3].

The patella frequently dislocates laterally due to an abrupt twisting motion or direct impact with an externally rotated foot, typically without medical evaluation or intervention. While most dislocations tend to reduce spontaneously before medical evaluation is needed, they often recur and lead to patellofemoral instability (PFI) and arthritis. Chronic dislocations may allow patients to self-reduce injuries, which often involve tears of the medial patellofemoral ligament (MPFL) that help keep the patella stable medially and laterally; both these ligaments help provide medial/lateral support, respectively, for patella stability [4,5].

Patellar instability (PI) is when the patella abnormally dislocates or partially dislocates from its position in the patellofemoral joint. This condition can result from various factors such as injury, chronic looseness of ligaments, misalignment of bones, connective tissue disorders, or anatomical irregularities. People with PI often suffer from intense pain, limited function, and an increased risk of long-term arthritis. Patellar dislocations (PD) make up about 3% of all knee injuries and are more common in young individuals, particularly females aged 10-16. Although the overall incidence of PI is 5.8 per 100,000 people, it is significantly higher in 10-17-year-olds, with 29 out of every 100,000 experiencing this issue [6,7].

Initial treatment for PD that does not involve loose bodies or damage to the joint typically consists of conservative methods. However, the recurrence rate for these initial dislocations ranges from 15% to 44%. Patients with two or more dislocations have a 50% chance of experiencing further episodes. A previous PD is a significant risk factor for ongoing instability, and this risk increases substantially if an MRI confirms an injury to the MPFL. First-time dislocators often continue to experience pain, functional limitations, and instability despite initial treatments [8,9].

The history of diagnosing and surgically treating misalignment of the knee extensor mechanism and PI has shown significant progress. Early approaches involved physical examinations and patient accounts for diagnosis, and initial treatments included immobilization and rest as the primary strategies. The widespread use of X-ray technology during the late 19th and early 20th centuries enabled more precise assessments of bone alignment and fractures. Arthroscopy, introduced in the mid-20th century, transformed the diagnosis and treatment of knee injuries by allowing direct visualization of the joint without significant invasiveness. Advanced surgical techniques, including tendon repair and reconstruction methods, were also developed to restore alignment and function. In recent decades, there have been major advances in medical imaging technology, with MRI scans providing detailed images of soft tissues, and minimally invasive surgical approaches that improve outcomes and shorten recovery times. These advancements significantly depart from early methods to today's precise and effective diagnostic and surgical interventions [10,11].

## Review

Diagnostic tools for patellofemoral instability

The common history of an extensor tendon rupture often involves a minor injury, typically indirect, resulting from a sudden muscle contraction while trying to avoid a fall. This is usually felt as severe pain. The clinical diagnostic trio includes sudden pain, the inability to extend the knee actively, and a noticeable gap at the rupture site [12].

In instances of a quadriceps tendon rupture (QTR), the extensor mechanism may still partially function due to an intact retinaculum and iliotibial tract, allowing for straight leg raising. A posttraumatic hematoma, particularly with delayed presentation, might obscure the noticeable gap. Clinical signs of QTR may also involve a low-riding patella (patella baja). Conversely, in a patellar tendon rupture (PTR), the noticeable gap is more visible due to the smaller amount of surrounding soft tissue. During examination, a high-riding patella (patella alta) is a common discovery, and it does not follow the tibia during knee flexion. Although MRI is considered the superior imaging method, it rarely provides additional information that would alter the treatment plan. It is more expensive and less accessible than ultrasound (US) but may offer further information in chronic cases regarding tendon quality, muscle atrophy, or fatty degeneration [13].

Additionally, a study conducted by McKinney et al. uncovered a notably high occurrence of associated intra-articular knee injuries, with 9.6% in QTR and 30% in PTR [14]. The most common were tears in the anterior cruciate ligament (18%) and medial meniscus (18%), especially in high-energy trauma situations. Therefore, MRI should be considered for younger patients with high-risk injury mechanisms [14].

The tibial tuberosity-trochlear groove distance: a traditional diagnostic method

The distance between the tibial tuberosity-trochlear groove (TT-TG) is commonly used to evaluate the lateral force vectors contributing to patella instability. Steenson et al. conducted a comprehensive MRI analysis on 60 patients diagnosed with PFI compared to 120 control subjects and found no statistical difference [15]. Researchers found that patients with repeated PD had higher instances of patella alta, increased TT-TG distance, torsional deformity, and trochlear dysplasia than those without such histories. This study emphasized anatomical factors contributing to PD without suggesting that an elevated TT-TG distance warranted surgical intervention via TTO [15].

Dejour et al. conducted a landmark CT scan study to measure TT-TG distance in fully extended knees, comparing patients with PI to unaffected controls [16]. Measurements were taken by drawing a perpendicular line through the deepest point of the trochlea to a transverse line passing from behind the posterior end of the femoral condyles and another vertical line from this to the center of the most anterior part of the tibial tubercle. The mean TT-TG distance in the PI group was 19.8 mm (standard deviation: 1.6), while it was 12.7 mm (SD: 3.4) in the control group (p<0.01). They identified a pathological threshold of 20 mm; 56% of instability group members exceeded this value versus only 3.5% of controls. Although some surgeons consider a TT-TG distance greater than 20 mm as the minimum criterion for distal realignment procedures designed to prevent recurrent instability, measuring this groove may prove more challenging in cases with trochlear dysplasia, which is common among these patients [15,17,18]. An increased TT-TG value should not immediately lead to osteotomy and medialization of the tubercle; instead, an in-depth evaluation for dysplasia should occur first, with deepening trochleoplasty potentially necessary to decrease lateralized force vectors. Dejour et al. advocated for medializing the TTO to reduce its distance to 10-15 mm, while Koeter et al. suggested 15 mm as the threshold for considering medializing TTO procedures [16,19].

According to Vairo et al., when measured using MRI, the TT-TG distance is a reliable and accurate indicator of PFI. Their study primarily aimed to analyze the interrater reliability of TT-TG distance among orthopedic surgeons with varying experience levels. It found excellent reliability with an intraclass correlation coefficient of 0.93 for PFI patients and 0.95 for controls. Additionally, the study compared TT-TG distances between PFI patients and controls, revealing that PFI patients had a significantly greater mean TT-TG distance (14 mm) than controls (10 mm). They identified a TT-TG distance threshold of 13 mm indicative of PFI risk, with this measure being two times more likely to be found in PFI patients. However, the authors caution that clinical decision-making should not rely solely on this criterion and should consider other relevant factors [20].

While the TT-TG distance helps assess the lateral force vector contributing to patella instability, it has limitations. Measurements can vary depending on the imaging modality and technique, leading to inconsistencies. In patients with trochlear dysplasia, the groove can be too intricate to measure accurately, potentially leading to erroneous assessments. TT-TG is a static measurement and does not account for dynamic factors affecting patellar stability, such as muscle function and ligament laxity. The pathological limits for TT-TG should not be universally applicable due to the anatomical variations between populations and individuals. Relying solely on TT-TG to decide on surgical interventions, such as tibial tubercle osteotomy, can be problematic as PI is often a multifactorial pathology. High TT-TG values alone should not automatically lead to surgery, comprehensive clinical evaluation is crucial. Additionally, TT-TG distance can change after surgical interventions, complicating postoperative evaluation. Accurate measurement requires technical expertise, which may not be uniformly available [20].

Emerging diagnostic parameters

In a research conducted by Li et al., they evaluated the dependability of the TT-TG distance in assessing tibial tubercle lateralization (TTL) in individuals with PD. The study comprised 96 PD patients and 96 control individuals who all underwent computed tomography (CT) scans. The researchers measured various parameters, including TT-TG, tibial tubercle-midepicondyle (TT-ME), tibial tubercle-Roman arch (TT-RA), tibial tubercle-tibial intercondylar midpoint (TT-TIM), and tibial tubercle-mid inter-epicondyle trochlea intersection (TT-MIELTI) distances. They discovered that all parameters, except TT-TIM, were notably larger in the PD group compared to the control group. TT-TG exhibited the strongest correlation with TTL. The study revealed excellent consistency among measurements, indicating high reliability. Particularly, the TT-TG distance displayed a moderate correlation coefficient (r = 0.601) with TTL, establishing it as a dependable measure of TTL in PD patients [21]. Moreover, the PD group's TT-TG, TT-ME, TT-RA, and TT-MIELTI distances were significantly larger, emphasizing their importance in evaluating PI. Despite introducing new parameters, the researchers concluded that the TT-TG distance remains a highly reliable metric for assessing TTL in PD patients. Although TT-ME, TT-RA, and TT-MIELTI also exhibited significant differences between groups, TT-TG demonstrated the strongest correlation with TTL. The study underscores the significance of utilizing TT-TG in clinical assessments and surgical decision-making for PI [21].

When comparing the different measurements, the study found that TT-TG was the most correlated with TTL, making it the superior parameter for assessing TTL in PD patients. While TT-ME and TT-RA distances also displayed significant correlations, they were less pronounced than TT-TG. The TT-MIELTI distance was reliable but did not exceed TT-TG in reflecting TTL. Interestingly, TT-TIM did not exhibit a significant difference between PD and control groups, indicating its limited utility. These findings support using TT-TG distance as the most effective measure for evaluating TTL [21].

In another study by Dai et al., they compared the effectiveness of various methods for measuring TTL in individuals with PFI. The study analyzed 47 PFI patients and 47 control individuals using CT and magnetic resonance imaging (MRI). They assessed the T-TG distance using conventional CT, three-dimensional CT reconstruction (TDR-TT-TG), and tibial tubercle-posterior cruciate ligament (TT-PCL) distance using MRI. The findings demonstrated high consistency between TT-TG and TDR-TT-TG measurements (ICCs of 0.852 and 0.864, respectively) and good reliability for TT-PCL distance (ICC of 0.758). The similarity between TT-TG and TDR-TT-TG measurements suggests that TDR-TT-TG is a viable alternative for evaluating TTL [22].

All three methods, TT-TG, TDR-TT-TG, and TT-PCL distances, were notably higher in PFI patients than controls. The mean TT-TG and TDR-TT-TG distances in PFI patients were 19.0 mm, while the TT-PCL distance was 25.1 mm. Bland-Altman analysis indicated good agreement between TT-TG and TDR-TT-TG measurements, suggesting that TDR-TT-TG is interchangeable with TT-TG for assessing tibial tubercle position. This finding supports the potential of three-dimensional CT reconstruction for reliable measurements in surgical planning for PFI cases [22]. Comparing the different measurements, the TT-TG distance remains the most correlated with TTL and is the most influential parameter for assessing TTL in individuals with PD. While TT-ME and TT-RA distances also displayed significant correlations, they were less robust than TT-TG. The TT-MIELTI distance, although reliable, did not surpass TT-TG in reflecting TTL. Interestingly, the TT-TIM distance did not significantly differ between PFI and control groups, suggesting its limited utility. These comparisons reinforce the TT-TG distance as the most effective measure for evaluating TTL, supporting its continued use in clinical practice [22]. These comparisons reinforce the TT-TG distance as the most effective measure for evaluating TTL, supporting its continued use in clinical practice [22].

In another study by Carrillon et al., the lateral trochlear inclination (LTI) was evaluated as a diagnostic tool for PI using MRI. The study involved 30 patients with PI and 30 control patients with nonspecific internal knee derangement. The results indicated that the LTI values differed significantly between the two groups, with a threshold value of 11° highly indicative of PI. The LTI measurement demonstrated excellent reproducibility, with an intraclass correlation coefficient exceeding 0.98. The study concluded that LTI is a reliable and accurate diagnostic tool for identifying PI [23].

Role of 3D imaging and its impact on diagnostic precision

Remember the following text. TEXT: Various imaging methods are used to diagnose PFI, including radiographic exams, CT scans, and MRI. Radiographs are essential for ruling out loose bodies in the medial patellar facet or lateral femoral condyle. Anteroposterior (AP) radiographs provide an overall analysis of lower extremity alignment, while lateral radiographs assess patellar height using ratios such as Insall-Salvati, Blackburne-Peel, and Caton-Deschamps at 30° of knee flexion [24].

Radiographs also help assess patellar tilt and trochlear dysplasia, and specific signs like crossing signs and double contour signs can be seen on lateral radiographs, indicating flattened trochlear grooves or hypoplastic medial femoral condyles, respectively. Sunrise/merchant views are particularly effective at identifying patellar tilt and the lateral patellofemoral angle; congruence angles serve as indicators of subluxation, with greater positive angles indicating more significant subluxation [25,26].

CT scans provide detailed assessments of femoral anteversion and rotation and measure the tubercle to TT-TG distance, which should not exceed 20 mm, an abnormality strongly suggesting PI. MRI is especially beneficial for identifying loose bodies, osteochondral lesions, and bone bruises on the medial patellar facet and lateral condyle surfaces, as well as MPFL injuries at their origin, abductor attachment, and midsubstance [16,27].

Three-dimensional imaging plays an integral part in diagnosing PI. By offering an in-depth view of knee anatomy and providing accurate assessment, it is an unrivaled assessment tool. Carrillon et al. have found MRI's measurement of LTI, an effective technique for diagnosing femoral trochlear dysplasia, a common factor contributing to PI, to be highly accurate at measuring LTI values; its excellent reproducibility and accuracy make its use and differentiates patients suffering from nonspecific internal derangements [23,28,29].

As a general limitation of conventional radiography, which often fails to detect small dysplasias in the proximal portions of the trochlea, three-dimensional MRI, by contrast, provides a clear and complete visualization of the patellofemoral joints (including the proximal trochlea), making MRI-based diagnosis much more precise; it is particularly advantageous in cases when clinical examination and conventional imaging results prove inconclusive and subclinical forms of PI are identified and managed efficiently [23,30].

Three-dimensional imaging is crucial for diagnosing PI, offering an in-depth view of knee anatomy and providing accurate assessment. Carrillon et al. found MRI's measurement of LTI to be highly accurate at diagnosing femoral trochlear dysplasia, and three-dimensional MRI provides a clear and complete visualization of patellofemoral joints, making MRI diagnosis much more precise [31].

Three-dimensional imaging also extends into surgical planning and evaluation of treatment outcomes, allowing surgeons to plan precise interventions tailored to individual patient anatomy for improved surgical results. Astur et al.'s study underscores the value of three-dimensional imaging in visualizing relationships among the quadriceps muscle, patellar tendon, and surrounding ligaments, offering depth perception that enhances anatomical studies and diagnostics [32].

Advanced imaging technology is essential for diagnosing and treating PI, as traditional methods often fail to capture the complete anatomical picture. Three-dimensional imaging allows for detailed anatomical visualization, revealing subtleties and providing a clearer view of patellar tracking within the trochlear groove [33]. Carrillon et al. demonstrated that measuring LTI using MRI significantly improves the accuracy of diagnosing PI and planning surgeries [23].

Beyond diagnostics, 3D imaging is invaluable for surgical training and preoperative planning. Surgeons can use 3D models to simulate procedures, understand a patient's unique anatomy, anticipate challenges, and improve precision, reducing the risk of complications. Integrating 3D imaging into both diagnostic and therapeutic protocols represents a significant advancement in managing knee-related disorders, specifically extensor mechanism dysfunction [32].

Treatment methods for patellofemoral instability

Nonoperative treatments for PI include closed reduction, NSAIDs, activity modification, and physical therapy, particularly for first-time dislocations, cases without loose bodies or articular damage, and patients with connective tissue diseases like Ehlers-Danlos syndrome. Physical therapy should emphasize closed chain exercises, quadriceps strengthening, and core and gluteal muscle strengthening to improve hip external rotation and decrease the Q-angle. Patella sleeves and patellar taping can also be beneficial [25,34].

Operative interventions are considered for osteochondral injury with loose bodies, chronic instability, and when nonsurgical treatments fail. Arthroscopic debridement is used to remove loose bodies identified on imaging. Open reduction internal fixation with screws and pins is performed if sufficient bone is available. MPFL repair is indicated for acute first-time dislocations with bony fragments, although its superiority over nonoperative treatment is not established. MPFL reconstruction with autograft or allograft is used for recurrent instability without malalignment or trochlear dysplasia, often utilizing gracilis and semitendinosus tendons [35].

A Fulkerson-type osteotomy, involving anterior and medial tibial tubercle transfer, is used for patellofemoral maltracking with degenerative changes on the distal and lateral patella, especially when TT-TG exceeds 20 mm. This procedure reduces pressure on the lateral patellar facet and trochlea but shows poorer outcomes with Outerbridge grade 3 or 4 lesions and is likely to fail with large central or medial trochlear lesions or diffuse patellar arthritis [36,37].

Arthroscopic surgery in patellofemoral instability

Arthroscopic surgery is extremely useful in treating patellofemoral instability, offering diagnostic and therapeutic benefits. This minimally invasive method allows for a detailed visual examination of the joint to identify structural issues contributing to instability, such as trochlear dysplasia, patellar tilt, or soft tissue abnormalities. Arthroscopy enables targeted interventions to address these issues with minimal disruption to surrounding tissues [38].

The "lateral release procedure" is a common arthroscopic procedure to correct excessive lateral patellar tilt. It aims to address tightness in the lateral retinaculum and release any tight structures to help reposition the patella more medially and improve its tracking through the trochlear groove. Arthroscopic MPFL reconstruction can also be performed to stabilize patella dislocation, often using autografts or allografts to restore function and increase patellar stability [35,39].

Arthroscopic techniques also allow for the efficient removal of loose bodies and the repair of osteochondral injuries in cases of PFI. This helps relieve discomfort while protecting cartilage from damage, crucial when treating active young patients at risk of chronic instability and osteoarthritis if left untreated. Arthroscopic surgery is essential in the management of PFI, providing minimally invasive solutions with reduced recovery times and postoperative discomfort compared to open surgeries. Additionally, it offers enhanced visibility, enabling more precise interventions and leading to improved clinical results. These advantages not only address immediate symptoms of instability but also help prevent long-term complications, ultimately improving the quality of life for patients (Table 1) [40].

**Table 1:** Advantages, Disadvantages, and Clinical Outcomes of Arthroscopic Surgery for Patellofemoral Instability PFI, patellofemoral instability

Aspect	Advantages	Disadvantages	Clinical Outcomes
Minimally invasive	Smaller incisions lead to less postoperative pain. Reduced risk of infection. Shorter hospitalization and faster recovery.	Requires a high level of surgical skill. Limited visualization and access compared to open surgery.	Faster return to normal activities. Reduced postoperative discomfort and complications.
Precision	Enhanced visualization of the joint with arthroscope. Precise identification and treatment of pathology.	Potential for technical difficulties. The learning curve for surgeons	Improved accuracy in diagnosing and treating instability. Higher satisfaction rates in skilled hands.
Rehabilitation	Early mobilization and weight-bearing possible. Accelerated rehabilitation protocols.	Requires strict adherence to rehabilitation protocols to avoid recurrence or complications.	Quicker return to functional activities and sports. Lower risk of joint stiffness post-surgery.
Complications	Lower risk of infection and complications such as blood clots and wound issues. Reduced risk of iatrogenic damage to surrounding tissues.	Potential for specific arthroscopy-related complications such as fluid extravasation. Requires careful intraoperative management.	Overall lower complication rates compared to open surgery. Better preservation of surrounding anatomy.
Long-term stability	Effective in addressing the underlying causes of instability. Potential for improved long-term stability and function.	Risk of recurrence if not properly performed. Long-term outcomes dependent on the correct identification and treatment of all contributing factors.	Improved stability and function with proper technique. Lower recurrence rates in properly selected cases.

Tanaka et al. conducted a study demonstrating the ability to detect significant changes in patellar lateralization and patellotrochlear distance in intact and medial patellofemoral complex (MPFC)-deficient knees using digitally-assisted arthroscopic measurements. They also found that these changes could be corrected with MPFC reconstruction. The MPFC consists of two important ligaments: the MPFL and the medial quadriceps tendon femoral ligament (MQTFL). These ligaments are crucial for the stability of the patellofemoral joint, particularly in providing medial support to the knee and preventing excessive lateral displacement of the patella. The MPFL primarily works to resist lateral patellar translation. At the same time, the MQTFL plays a significant role in distributing forces across the patella, contributing to the overall stabilization of the knee joint. These findings were particularly substantial at early flexion angles, with more than 7 mm of lateral overhang at 20-30° of flexion and more than 6 mm of patellotrochlear distance at 10-20° of flexion, indicating MPFC deficiency [41].

The MPFC has been shown to provide stability to lateral patellar translation at early flexion angles, with multiple biomechanical studies highlighting its role in limiting lateral patellar movement in response to lateralizing forces [42]. However, less has been described about the static position of the patella in the context of MPFC deficiency, particularly in the setting of arthroscopy. While abnormal patellar positions, such as increased lateralization and tilt, have been documented using static imaging like radiographs and MRI, descriptions of these abnormalities during arthroscopy remain limited [43,44].

Arthroscopic measurement techniques have not been widely described or utilized. Vaswani et al. recently detailed an arthroscopic measurement technique using an arthroscopic ruler, reporting reliable intraoperative measurements of the intercondylar notch that correlated well with MRI measurements [45]. Oakley et al. found that measurements' accuracy and interobserver reliability on knee arthroscopy, particularly for measuring lesion size, improved using calibrated probes with variable angles [46]. Aligning the probes with the lesion allowed for better visualization.

Future advancements in arthroscopic measurement tools and improved measurement methods that better capture changes in patellar tilt could broaden the application of our findings to intraoperative assessments of the patellofemoral joint during surgical procedures. As assessing graft function during MPFC reconstruction remains challenging to quantify, further studies are recommended to explore the applicability of these findings to intraoperative evaluations during the surgical treatment of PI.

## Conclusions

In conclusion, significant advances in diagnostic and surgical techniques have profoundly enhanced our understanding and treatment of extensor mechanism misalignment in the knee. This comprehensive review highlights the pivotal role of three-dimensional imaging technologies, such as MRI and CT, which offer unprecedented precision in diagnosing anatomical abnormalities contributing to patellofemoral instability. Integrating these advanced imaging modalities facilitates more accurate assessments and enables personalized treatment plans tailored to each patient's anatomical and biomechanical needs.

Arthroscopic surgery has emerged as a minimally invasive and highly effective approach for addressing various components of knee extensor mechanism misalignment. Techniques such as lateral release, MPFL reconstruction, and tibial tubercle osteotomy have improved outcomes with reduced recovery times and lower complication rates than traditional open surgeries. Innovations in arthroscopic measurement tools and techniques, including digitally calibrated instruments, have refined surgical precision and intraoperative decision-making.

Continued advancements in imaging technology, personalized medicine, and minimally invasive surgical methods are likely to shape future directions in the management of patellofemoral instability.
